# What proportion of adult allergy referrals to secondary care could be dealt with in primary care by a GP with special interest?

**DOI:** 10.1186/s13601-016-0091-1

**Published:** 2016-01-21

**Authors:** H. E. Smith, J. Wade, A. J. Frew

**Affiliations:** 1 Division of Primary Care and Public Health, Brighton and Sussex Medical School, Room 319, Mayfield House, Falmer, Brighton, BN1 9PH UK; 2 Department of Respiratory Medicine, Royal Sussex County Hospital, Brighton, UK

**Keywords:** Allergy, GP with special interest, Referral letter, Commissioning, Adult patients

## Abstract

**Background:**

The concept of a General Practitioner with Special Interest (GPwSI) was first proposed in the 2000 National Health Service Plan, as a way of providing specialised treatment closer to the patient’s home and reducing hospital waiting times. Given the patchy and inadequate provision of allergy services in the UK the introduction of GPwSIs might reduce the pressure on existing specialist services.

**Objectives:**

This study assessed what proportion of referrals to a specialist allergy clinic could be managed in a GPwSI allergy service with a predefined range of facilities and expertise (accurate diagnosis and management of allergy; skin prick testing; provision of advice on allergen avoidance; ability to assess suitability for desensitisation).

**Methods:**

100 consecutive GP referrals to a hospital allergy clinic were reviewed to determine whether patients could be seen in a community-based clinic led by a general practitioner with special interest (GPwSI) allergy. The documentation relating to each referral was independently assessed by three allergy specialists. The referrals were judged initially on the referral letter alone and then re-assessed with the benefit of information summarised in the clinic letter, to determine whether appropriate triage decisions could be made prospectively. The proportion of referrals suitable for a GPwSI was calculated and their referral characteristics identified.

**Results:**

29 % referrals were judged unanimously appropriate for management by a GPwSI and an additional 30 % by 2 of the 3 reviewers. 18 % referrals were unsuitable for a GPwSI service because of the complexity of the presenting problem, patient co-morbidity or the need for specialist knowledge or facilities.

**Conclusions and clinical relevance:**

At least a quarter, and possibly half, of allergy referrals to our hospital-based service could be dealt with in a GPwSI clinic, thereby diversifying the patient pathway, allowing specialist services to focus on more complex cases and reducing the waiting time for first appointments.

## Background

In the UK it has been proposed that a proportion of allergy referrals to specialists in secondary care could in fact be handled in the community by a GP with special interest (GPwSI) in allergy [1, 2]. There is no standard definition of the skills and facilities needed in such a service, but they might include: expertise in accurate diagnosis and management of allergy; provision of advice on allergen avoidance; ability to assess suitability for desensitisation; skin prick testing (SPT) to common airborne allergens; specific IgE blood tests; spirometry; and good links to local specialist services for managing patients whose problems turn out to be more complex than initially thought (i.e. beyond the scope of the GPwSI service).

The GPwSI concept was first proposed in the 2000 National Health Service Plan [3], as a way of providing specialised treatment closer to the patient’s home and reducing hospital waiting times. In essence a GPwSI is first and foremost a generalist, but has specific skills in a particular area which enable them to deliver a specialised clinical service without the direct supervision of a certified specialist. The concept of GPwSI has been mentioned in subsequent Department of Health initiatives and policy documents, and broadened to include other professionals (specialist nurses, dentists, pharmacists) [4]. In this expanded context, the role is termed Practitioners with Special Interest (PwSIs).

Whilst the potential benefit of a GPwSI has been recognised no estimates exist of the proportion of referrals to a secondary care allergy clinic that could be dealt with in a GPwSI allergy service with a practitioner with a predefined range of facilities and expertise. This study addresses this defect.

## Methods

We reviewed 100 consecutive referrals from general practice of patients with suspected allergic problems to a hospital-based specialist in allergy and respiratory medicine. Referrals from other specialists or hospitals were excluded from the study. Patient details, referring clinician characteristics, degree of urgency, main presenting problem, and reason for referral were analysed. The referral paperwork for each referral was anonymised and sent to three independent allergy specialists working in different hospitals for assessment. The reviewers were provided with a description of the knowledge, skills and resources of a potential GPwSI in Allergy: expertise in accurate diagnosis and management of allergic conditions (rhinitis, conjunctivitis, urticaria, angioedema, anaphylaxis, suspected food allergy); provision of advice (written and oral) on allergen avoidance; assessment of suitability for desensitisation; competence in performing and interpreting skin prick testing (SPT) to common airborne allergens; access to order and ability to interpret in vitro specific IgE blood tests. First, the referral letter was reviewed in isolation and a judgement made on whether it might be appropriate for a GPwSI consultation (suitable, not suitable, insufficient information to decide). The proportion judged suitable for a GPwSI consultation was calculated based on unanimous decisions (all three reviewers agreeing) and on the majority decision, where at least two out of three reviewers agreed. The demographic and clinical of the groups of patients judged suitable and unsuitable for GPwSI assessment were compared.

In order to ascertain whether prospective decisions of suitability for GPwSI referral were valid, reviewers were then invited to re-assess each referral again with the benefit of information summarised in the clinic letter. The degree of agreement of the two assessments, with and without additional data, was assessed using percentage agreement and Cohen’s kappa. As well as categorising suitability, reviewers were encouraged to make free text comments on their decisions and content analysis was used to identify key themes from this qualitative data.

## Results and discussion

### Characteristics of the referrals

The patients referred were aged 17–70 years old (median 33 years) and 77 % were female. The most common presenting clinical problems were food allergy (32 %) and angioedema (22 %) (Table 1). 92 % (92/100) of GP referral letters contained sufficient information for all of the reviewers to assess suitability for referral to GPwSI. One reviewer felt there was insufficient detail in 6 % (6/100) of the referral letters, one in 3 % (3/100) and the remaining reviewer considered all the letters adequate to make a triage decision.

**Table 1:** Characteristics of the patients referred (n = 100)

Mean age (median, range)/years	35.7 (33, 17 to 70)
Female	77 %
Primary reason for referral given in GP letter	
Diagnosis	31 %
Management advice	27 %
Test/investigation	42 %
Presenting clinical problem	
Food allergy	32 %
Nut allergy	17
Other food suspected	15
Angioedema	22 %
Urticaria	14 %
Asthma	10 %
Rhinitis	8 %
Drug allergy	4 %
Anaphylaxis	4 %
Other^a^	6 %
Tests and investigations performed in out-patient consultation^b^	
SPT	57
Serum specific IgE	25
Other blood tests	26
Imaging	4
No tests	12
Degree of urgency	
Urgent	3 %
Soon	3 %
Routine	92 %

^a^Other presenting problems included latex allergy, facial rash, hypereosinophilia, and cough

^b^Test and investigations do not sum to 100 as 23 patients had two or more tests

### Assessment of suitability

29 % of referrals were judged unanimously to be appropriate for management by a GPwSI (Table 2). Analysis of the presenting problems in this sub-group showed that the most frequent presenting problems considered suitable were suspected nut allergy and rhinitis (Table 3). An additional 30 % of referrals were judged as appropriate for GPwSI by 2 of the 3 reviewers (Table 2). In three of these cases, one of the reviewers felt there was insufficient detail to decide (i.e. no dissent), and in 27 two of the reviewers felt the referral was appropriate for GPwSI but one did not. These referrals were similar in terms of age, gender and presenting complaint to those with unanimous agreement of appropriateness.

**Table 2:** Opinions of three assessors on suitability for referrals to be seen in a GPwSI Allergy clinic—decisions based on referral letter alone

Opinion of assessors	Agreement between assessors	Number of referrals	95 % CI
Suitable for GPwSI	Full agreement (3/3 assessors)	29	21–39
	Partial agreement (2/3 assessors)^a^	30	22–40
Not suitable for GPwSI	Full agreement (3/3 assessors)	18	12–27
	Partial agreement (2/3 assessors)^b^	22	15–31
Insufficient information^c^		1	0–5
Total		100	

^a^Partial agreement in 3 of the cases arose because one of the three assessors felt there was insufficient information on which to make a decision

^b^Partial agreement in 4 of the cases arose because one of the three assessors felt there was insufficient information on which to make a decision

^c^2 reviewers felt there was insufficient information on which to assess suitability so no majority decision could be made

**Table 3:** Comparison of presenting clinical problem in referrals judged unanimously suitable or unsuitable for GPwSI assessment

Presenting clinical problem	No. judged suitable	No. judged unsuitable
Food allergy (nut)	12	0
Rhinitis	5	0
Anaphylaxis	2	1
Urticaria	3	2
Food allergy (other)	3	4
Angioedema	3	4
Asthma	0	2
Drug allergy	0	2
Other	1	3
Totals	29	18

### Cases unsuitable for GPwSI assessment

18 % referrals were judged unanimously to be unsuitable for a GPwSI service (Table 2). An additional 22 % of referrals were judged as inappropriate for GPwSI by two of the three reviewers (Table 2). In four of these cases one of the reviewers felt there was insufficient detail to decide (i.e. no dissent), and in 18 two of the reviewers felt the referral was inappropriate for GPwSI but one did not. Analysis of the free text associated with the cases which were judged unsuitable for referral to a GPwSI service revealed five key themes; clinical complexity, history suggesting a non-IgE mediated allergic problem, requirement for specialist knowledge, need for a service not available through GPwSI, and patient co-morbidity (Box 1).

### Box 1: The five types of reasons given for unsuitability for GPwSI (each theme is followed by some verbatim quotes to illustrate)

- Complexity of problem
  - multiple food allergies, needs SPT & RAST, and may need supervised challenges
  - Sounds more than just simple angio-oedema
- History suggestive of non-IgE mediated allergic problem
  - Unlikely to be IgE mediated
  - Potentially non- IgE food symptoms
- Specialist knowledge
  - If IgE mediated, unlikely to be one of the major allergens therefore specialist knowledge needed for avoidance advice
  - Complex funding issue. Probably best–secondary care specialist
  - Hypereosinophilia reported by GP
- Needs service not available from GPwSI
  - Needs prick–prick testing
  - Will need dietetic advice also
  - Again issue is primary care’s services access to dietetic support
- Patients with significant co-morbidity
  - Multiple co-morbidity
  - Complicated by preceding history of ulcers
  - Has combination of allergy and rheumatological condition

#### Validity of assessment based on referral letters

Referral letters were generally sufficiently detailed to predict appropriateness (Table 2). Whilst individual reviewers did revise some of their decisions about suitability for a GPwSI in the light of the clinic letter (Table 4), intra-observer agreement was high (89–95 %) (Table 5) with Kappa values all in excess of 0.8 indicating near complete agreement [5]. No patients judged suitable for GPwSI consultation were subsequently judged unsuitable. However, for six patients the initial majority decision of unsuitability for GPwSI (based on the referral letter) was changed to a majority opinion of suitability for GPwSI after reviewing the clinic letter. Reviewers felt that the problems identified in clinic were less complex than was anticipated from the referral letter, for example they commented ‘was *clearer once history obtained’*; *‘turned out to be a simple question re allergic status’*.

## Discussion

This study provides supportive evidence for the establishment of a GPwSI allergy service. In the absence of a national definition of the skills and competences for a GPwSI in Allergy, we set out an operational construct which is consistent with locally developed models. Using these criteria and the opinion of three allergy specialists it seems that at least a quarter and possibly a half of patients being referred to a hospital-based secondary care allergy service could be managed by a GPwSI. The development of a GPwSI allergy service could therefore be used to diversify the patient pathway, reduce the waiting list and liberate capacity for consultant-led hospital outpatient services to focus on those patients with more complex problems that need specialist assessment, investigations and ongoing management.

**Table 4:** Opinions of three assessors on suitability for referrals to be seen in a GPwSI Allergy clinic-decisions based on information summarised in the clinic letter written post-consultation

Opinion of assessors	Agreement between assessors	Number of referrals	95 % CI
Suitable for GPwSI	Full agreement (3/3 assessors)	29	21–39
	Partial agreement (2/3 assessors)^a^	36	27–46
Not suitable for GPwSI	Full agreement (3/3 assessors)	14	9–22
	Partial agreement (2/3 assessors)^a^	16	10–24
No agreement	None^b^	5	2–11
Total		100	

^a^In these cases in which there were partial agreements, all assessors had felt there was sufficient information to make a decision

^b^There was no agreement in these 5 patients because one reviewer felt there was insufficient information in the clinic letter to confirm or refute their original assessment of these cases based on the referral letter alone

**Table 5:** Intra-observer agreement of appropriateness for GPwSI, based on comparison of decision made based on referral letter and on clinic letter

	Reviewer 1	Reviewer 2	Reviewer 3
% agreement	95 %	89 %	92 %
Kappa	0.894	0.759	0.840
95 % confidence interval	0.804–0.984	0.623–0.896	0.773–0.946

As this study is unique we have no comparative data. However, it utilised a similar method to that used to ascertain the proportion of GP referrals to a hospital respiratory medicine clinic that would be suitable to be seen in a GPwSI respiratory clinic [6]. Although in a different specialty, the proportion of patients considered suitable for GPwSI was similar to our study: there was unanimous agreement for 23 % and variable degrees of agreement for a further 35 %. This is an exploratory and original study that focussed on the opinion of the consultant allergists as these were the individuals from whom the referrals would be diverted. In future work it would be advantageous to also seek also the opinions of GPwSIs, to measure their confidence and competence to deal with the patients’ problems that may be referred to them. Whilst this study is UK based, the challenges of inadequate allergy services are not unique to our health service, and the increase in allergic disease is common to all developed countries. We need pan-European working to innovative ways of delivering allergy services and evaluation methods.

In the development of any alternative care pathway, care needs to be given to issues of patient safety. GPwSI do not provide the same breadth of clinical care as a consultant-led service but the intention is that they provide care of equivalent quality and outcome. Triaging patients from the referral letter was feasible with almost all referral letters (94 %) containing sufficient detail to make a decision of suitability for a GPwSI consultation. When cases were reviewed retrospectively, shifts in assessment of suitability were few and were all from ‘unsuitable’ to ‘suitable’, confirming that decisions based on the referral letter are unlikely to lead to inappropriately complex patients being deflected to the GPwSI. It is important to avoid this outcome as such patients having been seen first by their GP and then a GPwSI, would still have unresolved issues and require further consultation with a specialist.

Commissioning of health services is the process by which health needs of the local population are identified, priorities for investment are set and appropriate services are purchased. In its 2010 consultation ‘Liberating the NHS: Commissioning for patients’ the Department of Health proposed a revised form of commissioning led by consortia of GP practices so that commissioning decisions are underpinned by clinical insight [7]. While the changes underway demand of us new ways of working they also provide an opportunity to adapt, expand and create services, including those for Allergy, for the benefit of our patients. This could allow us to redesign our currently overstretched hospital-orientated system of care for allergic disorders into a more integrated service which can better address unmet needs. The present data, used in discussion with primary and secondary care colleagues, could inform evidence-based decisions on commissioning alternative patient pathways. To achieve beneficial change for our patients, appropriateness of care, not locus of care, needs to be the primary consideration.

If GPwSI services are developed in allergy, it will be important to evaluate them formally so that there is a body of evidence to support their continuation and growth [8]. Only a randomised controlled trial of allergy referrals comparing an established GPwSI service with a hospital consultant-led service could address whether these two models of care are equivalent with respect to process (waiting times) and patient outcomes (symptom control, quality of life, satisfaction) and resource use. In 2003 Kernick commented on the paucity of evidence about the cost-effectiveness of GPwSI services compared with standard hospital outpatient care [9] and the economic evidence base remains small. A randomised evaluation of a GPwSI Dermatology service found that patients referred to the general practitioner with special interest cost the NHS more for little difference in clinical outcome [10]. However, the authors argued that these increased NHS costs could be offset against improvements in access, patient satisfaction and waiting times. Although one might expect the costs of being seen in a GPwSI service should be less than those of a hospital-based service, the findings of higher costs in this GPwSI dermatology clinic were consistent with previous evaluations of outreach clinics and initiatives to shift care from secondary to primary care [11, 12].

Formal evaluation is also needed to confirm introducing an additional tier of provision does in fact reduce the pressure on specialist allergy services. If Roemer’s Law of provider induced demand holds true in this context the existence of a GPwSI allergy service might increase the demand for assessment of patients who are currently being managed in primary care without specialist involvement. We know that long waiting lists may inhibit GPs from referring cases, so with expanded services, referrals may increase.

There are several challenges to establishing a GPwSI allergy service. We know that allergy is underrepresented in the undergraduate medical curriculum [13] and there is little coverage in postgraduate training for general practice [14]. In a speciality where there are few specialists there is a risk that they become preoccupied with patient care and are unable to allocate time for training colleagues, thus exacerbating further the demands on their clinical expertise. To minimise burden on overstretched local allergy services, GPwSI training could be centralised through existing Allergy courses at diploma and masters level, with clinical apprenticeships in regional allergy centres. GPwSI could achieve their continuing professional development through the activities of specialist organisations, such as the British Society of Allergy and Clinical Immunology (BSACI) or the European Academy of Allergy and Clinical Immunology (EAACI). In return the increasing involvement of generalists in specialist societies will ensure that the Primary Care perspective is incorporated into patient pathways, guidelines and algorithms.

Further work is needed to crystallize the role of a GPwSI in Allergy. Since 2009 GPwSI in any given clinical area have to be accredited via competency frameworks developed jointly between the Royal College of General Practitioners, the Department of Health and the Royal College of Pharmacists. Framework development is a rigorous process led by a GP clinical lead and a group of stakeholders relevant to the specialty (royal colleges, consultants, specialist societies, GP educators, patient groups). Before approval each framework undergoes an iterative process of stakeholder consultation and amendment until it is ready for approval by the RCGP Professional Development Board and the College’s Clinical Innovation and Research Centre chair. There are 17 existing frameworks of core activities and competencies for pharmacists (PhwSIs) and/or GPwSIs. These include Respiratory Medicine and Dermatology, but the development of an Allergy framework is still in progress.

We have focused on just one aspect of the role of the GPwSI: in addition to providing readily accessible expert advice the GPwSI could also play an important role in the education of their primary care colleagues (general practitioners, nurses, pharmacists, health visitors), development of other community-based service, clinical and prescribing advice, clinical leadership and advice for the commissioning of allergy services. The role of the GPwSI must not stand alone, they need to develop good pathways and integrate patient care with their local consultant allergists and organ based specialists with an interest in allergy. In addition there are potential GPwSI roles beyond health services, for example liaising with schools and local restaurants.

## Conclusion

In conclusion, up to half of the allergy referrals to our hospital-based service could be managed by a GPwSI in a community-based clinic, thereby diversifying the patient pathway, allowing specialist services to focus on more complex cases and reducing the waiting time for first appointments.

## Abbreviations

BSACI: British Society of Allergy and Clinical Immunology
EAACI: European Academy of Allergy and Clinical Immunology
GP: general practitioner
GPwSI: general practitioner with special interest
PhwSI: pharmacist with special interest
PwSI: practitioner with special interest
SPT: skin prick testing
